# Non-Invasive Retinal Biomarkers for Early Diagnosis of Alzheimer’s Disease

**DOI:** 10.3390/biomedicines13020283

**Published:** 2025-01-24

**Authors:** Snježana Kaštelan, Antonela Gverović Antunica, Velibor Puzović, Ana Didović Pavičić, Samir Čanović, Petra Kovačević, Pia Antonia Franciska Vučemilović, Suzana Konjevoda

**Affiliations:** 1 School of Medicine, University of Zagreb, 10000 Zagreb, Croatia; 2 Department of Ophthalmology, Clinical Hospital Dubrava, 10000 Zagreb, Croatia; 3 Department of Ophthalmology, General Hospital Dubrovnik, 20000 Dubrovnik, Croatia; 4 Department of Pathology, General Hospital Dubrovnik, 20000 Dubrovnik, Croatia; 5 Department of Ophthalmology, Zadar General Hospital, 23000 Zadar, Croatia; 6 Department of Health Studies, University of Zadar, 23000 Zadar, Croatia; 7 Department of Ophthalmology, University Hospital Center Zagreb, 10000 Zagreb, Croatia

**Keywords:** Alzheimer’s disease, retinal biomarkers, early diagnosis, optical coherence tomography (OCT), OCT angiography, non-invasive imaging, neurodegenerative disorders

## Abstract

Alzheimer’s disease (AD) is a progressive neurodegenerative disorder of the brain associated with ageing and is the most prevalent form of dementia, affecting an estimated 55 million people worldwide, with projections suggesting this number will exceed 150 million by 2050. With its increasing prevalence, AD represents a significant global health challenge with potentially serious social and economic consequences. Diagnosing AD is particularly challenging as it requires timely recognition. Currently, there is no effective therapy for AD; however, certain medications may help slow its progression. Existing diagnostic methods such as magnetic resonance imaging (MRI), computed tomography (CT), positron emission tomography (PET), and biomarker analysis in cerebrospinal fluid tend to be expensive and invasive, making them impractical for widespread use. Consequently, research into non-invasive biomarkers that enable early detection and screening for AD is a crucial area of contemporary clinical investigation. One promising approach for the early diagnosis of AD may be retinal imaging. As an extension of the central nervous system, the retina offers a distinctive opportunity for non-invasive brain structure and function assessment. Considering their shared embryological origins and the vascular and immunological similarities between the eye and brain, alterations in the retina may indicate pathological changes in the brain, including those specifically related to AD. Studies suggest that structural and vascular changes in the retina, particularly within the neuronal network and blood vessels, may act as markers of cerebral changes caused by AD. These retinal alterations have the potential to act as biomarkers for early diagnosis. Since AD is typically diagnosed only after a significant neuronal loss has occurred, identifying early diagnostic markers could enable timely intervention and help prevent disease progression. Non-invasive retinal imaging techniques, such as optical coherence tomography (OCT) and OCT angiography, provide accessible methods for the early detection of changes linked to AD. This review article focuses on the potential of retinal imaging as a non-invasive biomarker for early diagnosis of AD. Investigating the ageing of the retina and its connections to neurodegenerative processes could significantly enhance the diagnosis, monitoring, and treatment of AD, paving the way for new diagnostic and therapeutic approaches.

## 1. Introduction

Alzheimer’s disease (AD), the most common cause of dementia, manifests as a slowly progressive, irreversible neurodegenerative brain disease associated with ageing [1,2]. It is characterised by the loss of neurons in the brain, particularly in the cortex, leading to a gradual decline in behavioural, cognitive, and motor abilities. AD presents a significant diagnostic challenge, requiring early detection and intervention, and has emerged as a major public health issue [2,3,4]. Currently, over 55 million people live with dementia globally, with this number estimated to rise to 150 million by 2050 [5]. Early intervention is believed to slow the progression of AD, highlighting the importance of preclinical detection of neurodegeneration. Such early identification is essential not only for preventing AD-related dementia and advancing new treatment options but also for facilitating a personalised, patient-centred approach to care [6,7]. AD pathological changes in the brain are believed to begin decades before clinical symptoms appear [8]. In the clinical manifestation of cognitive impairment, the AD spectrum progresses from mild cognitive impairment (MCI) to overt AD dementia [9]. Interest in identifying new biomarkers for preclinical and clinical stages of the disease is motivated by the need for more accurate and refined diagnostic approaches and the necessity of identifying and recruiting patients for clinical trials focused on modifying disease progression in its early stages and assessing the efficacy of potential therapeutic regimens [1,7]. Early-stage detection for AD involves cognitive tests, neuroimaging techniques such as magnetic resonance imaging (MRI), functional MRI (fMRI), computed tomography (CT), positron emission tomography (PET), amyloid imaging, cerebrospinal fluid (CSF) analysis, blood tests, and genetic testing [10,11,12,13]. Despite these tools, a definite diagnosis can only be established through autopsy and neuropathological examination of brain tissue. Notably, post-mortem analysis has shown a misdiagnosis rate of up to 30% [14]. Current diagnostic methods are limited by low sensitivity and specificity, lack of standardisation, insufficient access to specialised imaging equipment, subspecialist expertise, and high costs [1]. In the absence of diagnostic procedures for the timely detection of AD onset, treatment options are ineffective since neuronal damage at that stage becomes irreversible. While disease-modifying therapy remains unavailable, certain drugs can help slow or alleviate some symptoms. This underscores the importance of early symptom recognition and timely initiation of treatment [2,15,16,17].

Diagnosing AD is particularly challenging, especially in its early stages. One major difficulty is that clinical symptoms typically manifest in the later stages of the disease, making early diagnosis complex and often delayed [18]. Additionally, the symptom overlap with other neurodegenerative diseases complicates differentiation, and available biomarkers may lack sufficient specificity or be present in individuals without AD. Accurate and widespread diagnosis is further complicated by limited access to specialised testing, ethical concerns, and economic issues [18]. As an extension of the central nervous system (CNS) and a potential window into the brain, the retina offers a unique opportunity to study the pathophysiology of many ophthalmological and neurodegenerative diseases. A growing body of evidence shows that both the brain and retina are affected by AD, and these pathological changes are significantly correlated [19,20,21]. Retinal changes are one of the earliest signs of AD, and several post-mortem studies have shown amyloid beta (Aβ) plaques and tau deposits in the retina of AD patients [19,22,23]. Some evidence also demonstrates that retinal nerve fibre layer (RNFL) thinning, which reflects the loss of retinal ganglion cells (RGCs), is significantly associated with brain atrophy. These changes have been confirmed using optical coherence tomography (OCT) and OCT angiography (OCTA) [24,25]. In addition to neurological deficits, evidence has shown that vascular factors also play a key role in the onset and progression of AD [26]. Advancements in technology have enabled the non-invasive visualisation and measurement of retinal microvasculature using OCTA. Unlike conventional colour fundus photography (CFP), OCTA provides visualisation of the retinal microvasculature at the micrometre level [7].

OCT, OCTA, and scanning laser ophthalmoscopy (SLO) represent promising tools for early AD diagnosis and assessing the risk of disease progression. In this review, we summarise recent advancements in non-invasive diagnostic techniques for retinal imaging, highlighting their utility in studying the pathophysiology of AD. Additionally, we explore the clinical implications of these findings and outline directions for future research to enhance our understanding and management of the disease.

A systematic review was conducted with a comprehensive literature search using the MEDLINE and PubMed databases up to December 2024. The search strategy incorporated a combination of the following keywords: “Alzheimer’s disease”, “retina”, “retinal biomarkers”, “optical coherence tomography”, “optical coherence tomography angiography”, “colour fundus photography”, “scanning laser ophthalmoscopy”, “retinal imaging”, “eye”, and “neurodegeneration”. The search was restricted to articles published in English. Duplicate records were removed, and full-text articles were obtained for all manuscripts relevant based on their titles and abstracts. The references of these full-text articles were reviewed to identify additional relevant studies that may not have been captured through the initial search strategy. Studies deemed significant to the research question were qualitatively assessed for inclusion, focusing on studies conducted within the last 15 years to ensure the review reflects current advancements in the field. No quantitative data synthesis was performed, as the aim was to provide a comprehensive narrative discussion on the topic.

## 2. The Causes and Clinical Outcomes of Alzheimer’s Disease

The proportion of individuals aged 65 and older is steadily increasing in developed countries. Given the strong correlation between ageing and the prevalence of AD, the challenges associated with healthcare for those affected by AD are anticipated to rise in the coming years. Notably, AD occurs approximately twice as frequently in women as in men; at age 65, the incidence of AD is 0.6% in men and 0.8% in women. The frequency of the disease escalates with age. It is estimated to increase by about 0.5% for each year between ages 65 and 69, approximately 1% for each year from ages 70 to 74, 2% for each year from ages 75 to 79, 3% per year from ages 80 to 84, and 8% per year for individuals aged 85 and older [27].

The exact cause of AD remains unknown; however, genetic factors are known to contribute, with an estimated 5% to 15% of cases exhibiting a familial link. Several specific genetic abnormalities are implicated in the disease. Some of these abnormalities can be inherited from just one parent, indicating that the abnormal gene is dominant. An affected parent has a 50% chance of passing this gene to each child, and about half of those children may develop AD before the age of 65 [28]. One notable genetic abnormality involves apolipoprotein E (Apo-E), a protein component of certain lipoproteins that transport cholesterol in the bloodstream. The gene encoding Apo-E has three major alleles, *Apo-E2*, *Apo-E3*, and *Apo-E4*, with *Apo-E4* being the strongest genetic risk factor for late-onset AD. Individuals carrying one or two copies of the *Apo-E4* allele have a higher risk of developing AD than those with the *Apo-E3* allele, while *Apo-E2* appears to have a protective effect. *Apo-E4* is associated with earlier onset and a more rapid disease progression [28]. However, it is important to note that genetic testing for the *Apo-E* type cannot predict whether an individual will develop AD, and such testing is not routinely recommended. In individuals with AD, pathohistological examinations of the brain reveal damage to the basal nucleus, which is located in the brain stem and produces the neurotransmitter acetylcholine, essential for transmitting impulses between brain cells. Effective communication between these cells is crucial for memory function, and the absence of acetylcholine significantly obstructs memory formation and retrieval [29].

AD manifests as progressive cognitive and neurological decline. As the disease advances, challenges may arise in various cognitive domains, including language, problem-solving, and spatial awareness [3]. In the early stages, individuals typically exhibit short-term memory loss, difficulty acquiring new information, and disorientation regarding time and place. Language impairments, such as difficulty finding words, and problems in judgement and decision-making may also be present. Additionally, mood changes, including anxiety and depression, can occur. As the disease progresses, memory loss intensifies, and patients may struggle to recognise familiar faces or recall significant aspects of their personal history. Behavioural changes, such as agitation, wandering, and aggression, become more common, alongside an increasing reliance on caregivers for daily activities. Communication abilities decline further, and sleep disturbances frequently occur. In the late stage, individuals lose nearly all language capabilities and require complete assistance with daily living. Severe motor dysfunction manifests as difficulties in walking and swallowing. Prominent physical symptoms include muscle rigidity, significant weight loss, and incontinence, reflecting the profound neurological decline that characterises the final stages of AD [30,31].

In patients with AD, the accumulation of Aβ deposit, an abnormal, insoluble protein, is evident in brain tissue. This accumulation occurs due to the inability of cells to process and remove senile plaques. Additionally, elevated levels of tau protein are observed. While the presence of these abnormalities is a part of the natural ageing process, they are significantly more prevalent in individuals with AD. The relationship between these brain tissue abnormalities and the onset of AD remains unclear; it is uncertain whether they are causative factors for the disease or the result of an underlying issue that contributes to both dementia and these brain changes. Research has noted that the proteins Aβ and tau associated with AD share similarities with abnormal proteins found in prion diseases [32,33].

AD poses significant diagnostic challenges due to its heterogeneous clinical presentation and symptom overlap with conditions such as Parkinson’s disease, frontotemporal dementia, and MCI. Early-stage AD often manifests as subtle cognitive impairments, mood changes, and behavioural shifts, frequently mistaken for normal ageing or other disorders. The lack of a definitive biomarker complicates early detection, particularly in the preclinical phase when symptoms are minimal. These challenges highlight the importance of a comprehensive, multimodal diagnostic approach incorporating clinical assessment, advanced neuroimaging, genetic testing, and emerging biomarkers to enhance accuracy and timely intervention [10,14,17,27].

## 3. Correlation Between the Retina and Brain in Deducing Alzheimer’s Disease

As a developmental extension of the CNS, the retina shares numerous anatomical and physiological characteristics with the brain [34,35,36,37]. Due to this intrinsic connection, the visual manifestations of AD have attracted considerable interest, with the retina emerging as a potential diagnostic tool for early detection [35,38,39,40]. Although visual acuity often remains relatively preserved in AD patients, deficits in various other visual functions are common, raising questions about the mechanisms underlying these impairments [38,39,40]. As research continues to explore the intricate relationship between the retina and the CNS, the retina stands out as a promising, non-invasive window into CNS processes [34,35,36,41,42].

Structurally, the retina is acknowledged as an integral part of the CNS, originating from the neural tube during embryonic development [35,38,39,40,41]. This close relationship is exemplified by RGCs, whose axons extend through the optic nerve, directly linking the retina to the brain [19,35,37]. The characteristics of RGCs, including their response to neurotransmitters and susceptibility to degeneration, further support the concept of the retina as a valuable model for studying CNS disorders [38,39,40,41]. The retina and the brain share diverse populations of neurons, glial cells, and specialised vascular systems, making them similarly responsive to pathological processes [34,35,43]. Studies have shown that retinal pathologies, such as RGC degeneration and microvascular changes, often mirror those found in the brain, particularly in neurodegenerative diseases [19,39,40,41,42,43]. This close structural and functional relationship supports the retina’s potential as a tool in the study and early detection of CNS disorders [19,34,35,36,38,39,40,44]. Anatomically, the retina and the brain exhibit layered structures consisting of neurons, supportive glial cells, and a blood–retinal barrier that shares many similarities with the blood–brain barrier [34,35,37,43]. Detailed studies of the retinal microvasculature have highlighted its remarkable resemblance to the brain’s small blood vessels, prompting the hypothesis that retinal vascular changes may correlate with cognitive decline and an increased risk of AD [19,40,41,42,43]. The early loss of pericytes and reduced platelet-derived growth factor receptor-β signalling within the retinal microvasculature have been associated with AD-related vascular changes and cognitive decline [41]. The tightly regulated blood–retinal barrier serves a similar function to the blood–brain barrier, helping to maintain microenvironment stability that is critical for neuronal health [35]. Current studies also highlight the presence of metabolic and oxidative stress responses in the retina, which may mirror those found in the brain, suggesting a shared pathophysiological mechanism [34,45]. This link allows comparisons between retinal health and cognitive function, supporting the hypothesis that retinal assessments can identify underlying CNS conditions [34,35]. Research has demonstrated that measuring RGC density and RNFL thickness offers valuable insights into the extent of neurodegeneration in the brain [39]. Recent findings have highlighted significant retinal changes in patients with AD, particularly the accumulation of Aβ and tau proteins, key condition features [19,40,41,42,43]. Research has convincingly demonstrated the presence of Aβ plaques within the retinal layers, mirroring the plaque formation observed in the brain of AD patients [19]. Advanced imaging techniques, such as OCT, have revealed thinning of the RNFL and reduced retinal blood flow, indicative of ongoing neurodegeneration [19,43]. Importantly, the presence of Aβ in the retina is thought to precede detectable neurodegeneration in the brain, suggesting that these retinal Aβ deposits may serve as early biomarkers for preclinical AD [45]. Significantly, Aβ oligomers detected in the retina have been associated with the loss of RGCs and impairment in visual function, emphasising the importance of ongoing monitoring of retinal health to assess the progression of neurodegenerative diseases [45]. Moreover, the correlation between retinal Aβ deposits and cognitive decline underscores the potential of retinal imaging as a diagnostic tool for AD [19]. Histopathological examinations of post-mortem retinal tissues have identified Aβ deposits, particularly within the RGC layer. This finding suggests that retinal Aβ accumulation may reflect similar pathological processes occurring in the brain, potentially explaining the visual disturbances observed in patients with AD [19]. A comprehensive understanding of the relationship between retinal and cerebral Aβ loads could significantly advance the development of innovative methods for early diagnosis and continuous AD monitoring. Moreover, the potential use of retinal amyloid imaging as a surrogate biomarker presents a novel approach for screening at-risk populations and evaluating the efficacy of therapeutic interventions [19,45].

Neuroinflammation is a critical component of AD pathology, and recent findings indicate that the retina exhibits similar inflammatory responses. Elevated levels of pro-inflammatory cytokines and activated glial cells have been observed in the retina of patients with AD, mirroring the neuroinflammatory changes in the brain [34,36,41,46]. Studies have documented increased gliosis and microglial activation in the retina of individuals with AD, suggesting an early response to pathological changes that may precede neurodegeneration [34,36,37,41]. The retinal microvasculature plays a crucial role in these inflammatory processes, with alterations in vascular integrity observed alongside neuroinflammatory markers. Specifically, reduced capillary density and compromised blood–retinal barrier integrity have been associated with cognitive changes, reflecting similar cerebrovascular alteration in the brain [19,37,41,43]. These vascular abnormalities may lead to decreased perfusion and exacerbate cognitive decline, underscoring the interplay between retinal and cerebral health [19,40,41,42,43]. Furthermore, retinal imaging techniques have shown that patients with AD frequently exhibit increased vascular abnormalities, including alterations in fractal dimension and vessel calibre, which may serve as markers of disease progression [36,37,40,43]. This highlights the potential of retinal evaluation for diagnosing AD, monitoring vascular health, and elucidating the mechanisms linking vascular changes to AD pathology. The anatomical and physiological parallels between the retina and the CNS position the retina as a crucial area of research for understanding neurodegenerative diseases, particularly Alzheimer’s. The detection of Aβ and tau proteins in the retina, along with observed vascular and inflammatory changes, underscores the retina’s role in the underlying pathological processes of the brain [36,37,40,41,44].

## 4. The Role of Retinal Imaging in Alzheimer’s Disease Diagnosis

An encouraging area of research is investigating ocular changes in patients with cognitive decline and AD. Advanced imaging techniques have become valuable diagnostic tools in ophthalmology, enabling detailed assessments of the retina and providing useful insights into retinal thickness, structure, and vasculature. As research advances in exploring the implications of retinal health within neurodegeneration, it is essential to integrate these advanced imaging modalities to expand our understanding of retinal pathologies. Ongoing developments in retinal imaging technology can significantly improve our ability to diagnose, monitor, and potentially intervene in AD progression. Studies have identified several correlations between AD and ocular changes, including the accumulation of Aβ in the retina and lens, retinal vascular abnormalities, and retinal nerve damage, evidenced by the thinning of the RNFL [47,48,49,50]. These imaging techniques provide valuable information about the ocular status of AD patients, offering potential biomarkers for early diagnosis and disease monitoring [51,52].

### 4.1. Current Retinal Imaging Techniques

The advancement of retinal imaging techniques has significantly enhanced our understanding and diagnosis of various ocular and systemic diseases. These technologies enable detailed visualisation of retinal structures and vascular dynamics, facilitating the early detection of pathological changes. Current techniques range from non-invasive methods, such as OCT and CFP, to more comprehensive approaches such as fluorescein angiography or SLO with curcumin. Each method offers distinct advantages and limitations, providing clinicians with valuable tools to assess retinal health and its potential implications for neurodegenerative conditions (Table 1) [53,54,55,56].

**Figure 1:**
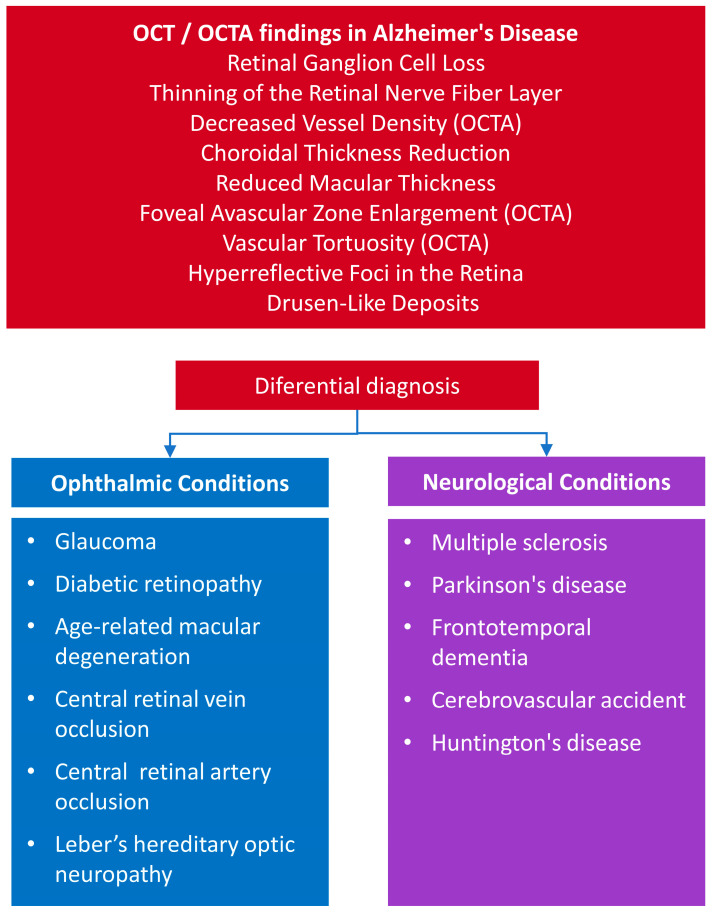
Differential diagnosis in OCT and OCTA findings in Alzheimer’s disease.

Today, the most commonly used imaging technique in ophthalmology is OCT, a non-invasive, non-contact tool capturing in vivo cross-sectional images of the retina, revealing its thickness and layered structure [57]. OCT provides valuable information regarding the nerve fibre layer surrounding the optic nerve head, enabling assessment of RGC axons and measuring RNFL thickness. It also evaluates the thickness of the RGC layer and the inner plexiform layer (IPL) in the macular region, which helps assess the RGC cell bodies and dendrites, known as the macular ganglion cell inner plexiform layer (GC-IPL) thickness. By integrating measurements of RNFL thickness and GC-IPL thickness, researchers can effectively analyse the macular ganglion cell complex (GCC) layer [53]. Additionally, OCT is crucial for visualising the overall macular thickness and provides two- or three-dimensional images, allowing for detailed analysis of the progression and rate of retinal changes over time. OCTA provides images of retinal vasculature without the need for invasive procedures or contrast agents, making it a safer, quicker, and more accessible option for patients and physicians. OCTA visualises retinal vasculature using motion contrast, detecting and highlighting movement from blood flow while excluding areas of the fundus without blood vessels [49]. This technique offers key insights into the surface area of blood vessels, including capillaries, known as vessel density. Additionally, OCTA allows for detailed visualisation of the fovea, the region with the highest concentration of cone receptors and oxygen consumption, but lacking capillaries [53,54]. OCT offers numerous advantages, including its ease of use, patient-friendly nature, and cost efficiency, making it a valuable tool for large-scale applications such as AD diagnosis. Its cost-effectiveness is particularly advantageous, as it enables examining many patients within a relatively short timeframe. As a non-invasive procedure, OCT is convenient for patients, requiring only 5–10 min to complete, with generally high compliance due to minimal patient participation. However, despite these benefits, OCT has limitations. It does not provide a comprehensive view of the orbit or detailed imaging of the retinal periphery [57]. While OCT and OCTA are routinely employed in ophthalmology, their application in AD diagnosis necessitates the validation of specific retinal biomarkers to distinguish AD-related changes from those caused by other conditions. Furthermore, developing cost-effective screening workflows is essential to ensure accessibility and scalability. Addressing these challenges will require continued technological advancements, targeted training programmes for clinicians and technicians, and robust large-scale validation studies. The feasibility and practicality of implementing OCT and OCTA as diagnostic tools for AD could be significantly enhanced by overcoming these barriers. Figure 1 illustrates differential diagnostic findings in OCT and OCTA images of patients with AD.

In the context of AD, OCT and OCTA have emerged as promising tools for detecting early retinal changes that may correlate with neurodegenerative processes. Significant structural alterations in the retina, particularly within the macular GCC, have been documented in AD patients. These changes are thought to reflect the underlying damage in the central nervous system, with a particular focus on the loss of RGCs. A substantial loss of GCC, observed in OCT imaging, could indicate a corresponding decline in the microcirculation responsible for supplying these cells. This suggests a direct link between retinal ganglion cell degeneration and impaired cerebral microvascular function, a key feature of AD pathology. OCTA provides detailed insights into the retinal vasculature, revealing alterations in vessel density and microcirculatory patterns that may be associated with AD progression. Specifically, a reduction in vessel density, particularly in the macular region, has been correlated with cognitive decline, suggesting that retinal microvascular changes could serve as an early biomarker of AD. Furthermore, impaired perfusion and reduced vascular complexity observed on OCTA may parallel the microcirculatory disturbances in the brain, providing a more comprehensive view of AD’s neurovascular implications [58,59,60]. The most detailed diagnostic method for retinal vasculature analysis is fluorescein angiography combined with CFP. This technique, which uses a contrast dye, provides critical information such as the full width of retinal arterioles and venules, including key metrics like the central retinal arteriole and central retinal venule equivalent. Vessel tortuosity, which reflects the overall health of the vasculature, can also be assessed [53]. This method is valuable for detecting haemorrhages, neovascularisation, and areas of non-perfusion. However, due to the use of contrast dye, it is invasive and provides only two-dimensional images [55,56].

SLO is a non-invasive imaging technique that uses a laser to scan the retina and generate high-resolution in vivo images. SLO has several advantages in retinal imaging, including its ability to produce detailed images of the retinal structures and detect subtle changes, such as the presence of amyloid deposits, particularly when fluorescent agents such as curcumin are used [61]. Curcumin, a natural compound known for its high affinity for Aβ aggregates, has been utilised in imaging studies to identify intraretinal amyloid deposits. The technique involves the oral or intravenous administration of curcumin, followed by imaging with SLO to visualise its fluorescence after binding to Aβ plaques. SLO is a high-resolution imaging modality that employs a laser beam to scan the retina, capturing detailed structural images. When combined with curcumin-based amyloid labelling, SLO effectively detects the fluorescent signal emitted by curcumin upon binding to Aβ plaques in the retina. This imaging technique offers a unique non-invasive opportunity to study AD-related changes by precisely visualising pathological deposits in the retina. One of the primary advantages of this method is its high specificity. Curcumin selectively binds to Aβ plaques, allowing for a targeted approach to identifying retinal changes associated with AD. Additionally, the non-invasive nature of SLO provides a valuable tool that complements other retinal imaging techniques, ensuring minimal patient discomfort. SLO’s high resolution is another key advantage, as it allows the detection of subtle retinal changes that may indicate the early stages of AD. This technique holds significant promise for early AD detection, as Aβ aggregation is one of the earliest events in the disease’s pathology. By identifying these changes at an early stage, it may be possible to diagnose preclinical AD before a more significant cognitive decline occurs. However, there are limitations to this approach that must be addressed. One major challenge is the bioavailability of curcumin, which is relatively low in its natural form. This may limit its effectiveness unless modified formulations or advanced delivery systems are employed to enhance its bioavailability. Additionally, variability in imaging protocols and curcumin formulations may pose difficulties in standardising the technique, potentially affecting the reproducibility and reliability of results. Finally, while the method shows considerable promise, further validation through large-scale clinical studies is required to establish its diagnostic utility and confirm its specificity for AD [62,63,64]. Alternatively, non-invasive CFP offers an in vivo image of the retinal vasculature without contrast application. It represents a widely accessible, cost-effective imaging technique to assess retinal vasculature. Recent advancements in image analysis algorithms have enabled the extraction of quantitative biomarkers, such as vessel calibre, tortuosity, branching angles, and fractal dimensions, which can reflect microvascular alterations associated with AD. These vascular biomarkers may correlate with cerebral microvascular dysfunction, providing indirect evidence of neurodegenerative changes in the brain. CFP’s advantages include its non-invasive nature, affordability, and broad availability, making it particularly suitable for large-scale population screening. Moreover, it does not require contrast agents, reducing the risk of adverse reactions. However, CFP has limitations. It offers lower sensitivity and resolution than advanced imaging techniques like OCTA. The lack of depth-resolved information can limit its ability to detect subtle microvascular changes in deeper retinal layers. Variability in image quality due to media opacities such as cataracts and challenges in standardising quantitative analysis across different devices and populations present further barriers to its widespread clinical use. Despite these challenges, integrating machine learning and artificial intelligence (AI) into CFP analysis holds promise for enhancing diagnostic accuracy and improving its potential for early detection of AD-related changes. Future studies should focus on validating these vascular biomarkers in longitudinal cohorts to determine their specificity and sensitivity for AD diagnosis and monitoring [7,65,66,67].

### 4.2. Retinal Biomarkers Associated with Alzheimer’s Disease

Diagnosing AD can be challenging when relying solely on clinical findings and family history. Recent studies have highlighted the potential of the retina as a valuable source for diagnostic measures in Alzheimer’s and other neurodegenerative disorders through its biomarkers [51,53,68,69,70,71,72,73,74,75] which can be categorised into three descriptive types: structural, vascular, and functional [46]. Each category provides unique insights into the underlying pathophysiology of AD, aiding in more accurate and timely diagnosis. The earliest AD signs and features are characterised by the deposition of Aβ plaques on the walls of blood vessels in the brain. A 2023 retrospective cross-sectional evaluative study on AD using OCT and OCTA compared AD with other forms of dementia, revealing the presence of hyperreflective foci linked to Aβ plaques in AD patients. Additionally, the study observed that the RNFL was thicker in AD patients than in those with other dementias, particularly in the early stages of the disease. These findings suggest that retinal imaging could offer valuable insights into the pathological changes of AD and may serve as an effective tool for the early differentiation of AD from other dementias [76]. These plaques form when the Aβ protein precursor is cleaved by various proteases, including beta and gamma secretases. The accumulation of Aβ fragments primarily occurs due to impaired clearance mechanisms, formatting rigid plaques that ultimately result in neuronal damage and cell death [77]. Notably, since the cerebral and retinal vasculature share similarities, Aβ is also known to deposit in the retina [48]. In addition to its presence on the walls of retinal blood vessels, Aβ aggregation has also been observed in the eye lens, which is particularly susceptible to degenerative changes. This protein build-up contributes to the lens-clouding characteristic of cataracts [52,78]. Regarding physical biomarkers, significant changes have been observed in the retina, particularly in its vasculature, optic disc, and RNFL. These alterations in the optic disc and retinal layers can be effectively visualised using OCT, while vascular changes can be assessed through OCTA, CFP, and fluorescein angiography [51,79]. As AD progresses, the gradual damage and eventual cell death lead to noticeable changes in the optic nerve head and the RNFL. Notably, patients with developing AD often exhibit a significantly increased cup-to-disc ratio and thinning of the RNFL [77,79,80]. Early stages of the disease are associated with decreased perfusion and areas of non-perfusion, particularly in the peripheral retina, alongside a reduced density of retinal microvascular networks compared to normal conditions. Even vessels without plaque accumulation may display venular tortuosity, adversely affecting blood flow quality. As the disease advances, changes become more pronounced in central areas, including the equatorial retina, eventually affecting the posterior pole [56].

### 4.3. Clinical Applications and Diagnostic Potential of Retinal Biomarkers

The challenge in diagnosing AD lies in the need for early detection of signs before cognitive decline becomes evident. While a positive family history may increase risk, the absence of clear clinical symptoms can complicate the diagnosis. Therefore, identifying Alzheimer’s biomarkers early is crucial for timely intervention and management of the disease. Detecting these biomarkers can facilitate a more accurate diagnosis and improve patient outcomes. Studies have demonstrated that changes in retinal structure and function, such as reductions in RNFL thickness and alterations in ganglion cell layer (GCL) metrics, may precede clinical signs of AD [81]. Established screening methods for detecting AD include PET imaging and the analysis of CSF biomarkers for Aβ and tau. However, these methods are often limited by their cost and invasive nature, and may not be readily available [77,82]. In this context, utilising the eye as a “window to the brain” presents an innovative alternative, allowing early AD detection through accessible and non-invasive retinal imaging techniques. Changes in retinal microvessels offer valuable insights into cerebrovascular health and AD. OCTA has been particularly useful for detecting these changes, with studies showing reduced retinal microvascular density in AD patients [54,83]. Notably, these microvascular alterations have also been observed in individuals with elevated Aβ levels detected by PET imaging or CSF assays, which are markers of preclinical AD [54]. Incorporating OCT into routine follow-up for patients at risk of AD could be highly advantageous with the predictive value and specificity of retinal biomarkers in identifying early signs. A meta-analysis found that reduced RNFL thickness is closely associated with the presence of amyloid plaques, a hallmark of AD pathology [73]. Table 2 summarises the role of OCT and OCTA changes and the challenges in the early diagnosis and monitoring of AD.

### 4.4. Challenges and Opportunities of Retinal Imaging for Alzheimer’s Disease Diagnosis

Retinal imaging via modalities such as OCT and OCTA has demonstrated the potential to identify AD-related biomarkers. However, translating this potential into a reliable clinical tool for AD diagnosis presents numerous challenges that must be addressed to ensure accuracy, reliability, and clinical utility. Factors such as small pupil size, cataracts, vitreous opacities, dry eyes, and eye movements can significantly compromise image clarity and resolution, hindering the capture of high-quality retinal images. In clinical practice, cooperation during the examination is one of the significant challenges in using OCT and OCTA for retinal imaging in AD patients. In advanced stages of AD, patients often experience cognitive and motor impairments, which can make it challenging to maintain proper gaze fixation during the imaging process. This lack of cooperation can result in suboptimal image quality and, consequently, affect the accuracy of the obtained measurements. Specifically, with OCTA, proper fixation is crucial for obtaining high-quality images of retinal vasculature. The inability to fixate the gaze, especially in advanced AD, may lead to motion artefacts, blurring, or distortions in the vascular images, compromising the accuracy of the resulting scan. These artefacts can negatively impact the processing and analysis of retinal vessel density, leading to an erroneous reduction in parameters critical for evaluating retinal health. This can result in an artefactual decline in vessel density measurements and potentially obscure meaningful changes related to the disease progression [84]. Inadequate pupil dilation can restrict the field of vision, making it difficult to detect subtle retinal changes associated with AD. While cataract formation is a typical ageing phenomenon, certain neurodegenerative processes are known to accelerate this progression, resulting in characteristic opacities. Several studies have identified Aβ aggregation, contributing to cataract formation [52,78,85]. On the other hand, many recent studies assessing the presence of AD-related proteins have been unable to replicate these findings, likely due to variations in laboratory techniques and differences in diagnostic criteria for AD [86,87,88]. However, regardless of the cause, cataracts, resulting from amyloid deposits or age-related degeneration, further diminish visual acuity by obstructing light transmission to the retina. Additionally, vitreous opacities can further degrade image quality, complicating the assessment of retinal biomarkers. The abnormal accumulation of amyloid fibrils in the retrolental space, referred to as retrolental amyloid deposition, can also significantly impede light transmission to the retina. This interference leads to reduced visual acuity and a diminished ability to detect fine retinal details, complicating the analysis of retinal biomarkers. The reduction in light passage impairs the high-resolution visualisation necessary for identifying early retinal biomarkers of AD, ultimately compromising the accuracy and reliability of early diagnostic evaluations [89,90]. These challenges highlight the need for careful consideration and optimisation of imaging techniques to ensure reliable screening results [79]. If a clear retinal image can be obtained, further considerations are necessary. Retinal biomarkers exhibit significant variability influenced by age, genetics, and lifestyle. Moreover, individuals with comorbidities such as diabetes, hypertension, obstructive sleep apnoea, age-related macular degeneration (ARMD), and glaucoma tend to exhibit more pronounced retinal changes than their healthier counterparts. These findings suggest that comorbid conditions may amplify the effects of AD on retinal structures, potentially complicating interpretation [77]. Retinal biomarkers exhibit distinct patterns associated with AD-related neurodegeneration, aiding in differentiation from other diseases and the normal ageing process. Integrating clinical assessments with neuroimaging data is essential for a comprehensive understanding. Therefore, carefully analysing these data is crucial to minimise the risk of false positives and negatives, as drawing objective conclusions remains a significant challenge [79].

## 5. Future Perspectives and Conclusions

AD is a neurodegenerative disorder marked by abnormal Aβ and tau protein accumulation in the brain. With rising global prevalence, AD presents major social and economic challenges. The need for reliable, accessible biomarkers is crucial to improve early detection, monitor disease progression, and assess treatment responses, ultimately enhancing clinical care and advancing therapy development [91]. The eye and brain share embryological origins, similar vascular systems, and immune functions, enabling non-invasive assessments of brain processes through the eye. Research shows that retinal changes, particularly in neuronal and vascular structures, reflect brain alterations and may serve as biomarkers for early dementia diagnosis. Further, abnormal accumulations of Aβ and tau proteins in AD patients’ RGC layer and inner retina were found, linking retinal pathology to AD. These retinal changes occur alongside similar changes in the brain, making retinal assessments a promising diagnostic approach for AD [2,18]. Although promising, many retinal biomarkers for AD are still in the early stages of validation in clinical settings. The transition from research findings to clinical application requires rigorous longitudinal studies, larger patient cohorts, and standardised imaging protocols to confirm their diagnostic and prognostic value. Moreover, integrating retinal biomarkers with established diagnostic tools, such as CSF analysis and neuroimaging, is essential to enhance diagnostic precision and relevance and their effective use in clinical workflows [18,24,38,51].

Early detection of AD through retinal biomarkers opens pathways for several targeted interventions. These include initiating personalised lifestyle modifications, such as adopting brain-healthy diets, regular physical activity, and cognitive training, which are known to mitigate the risk and slow disease progression. Additionally, timely initiation of emerging disease-modifying therapies, particularly those addressing Aβ and tau pathology, could significantly benefit from such early identification. Integrating retinal imaging into routine health assessments may also enable the stratification of patients for clinical trials, ensuring that therapeutic interventions are tested in individuals at preclinical or early disease stages, where they are likely to be most effective. Furthermore, a multidisciplinary approach combining ophthalmological and neurological care could provide a comprehensive framework for managing AD, potentially delaying cognitive decline and enhancing overall quality of life [3,9,15,17]. Table 3 outlines key recommendations for incorporating retinal biomarkers into AD research and diagnostic protocols.

Retinal imaging techniques such as OCT and OCTA are non-invasive, cost-effective methods with significant potential for research and clinical use. They are time-efficient and patient-friendly and have the potential for early detection and monitoring, identifying subtle retinal changes before cognitive symptoms of AD emerge [1,92,93,94]. Numerous challenges can impede the interpretation of findings, highlighting the need for careful consideration in future studies of retinal biomarkers. The diagnostic criteria for AD have evolved, potentially causing inconsistencies between patient populations diagnosed with AD and MCI based on clinical criteria versus those identified through stricter classifications relying on in vivo biomarkers such as CSF or PET/CT amyloid imaging. Furthermore, autopsy studies of patients with significant AD neuropathology have uncovered significant groups of individuals who do not display clinical symptoms of the disease. These discrepancies may contribute to unintentional misclassifications of patients and complicate efforts to generalise findings across different studies [1,95].

A limitation in retinal imaging for AD screening could be obtaining reliable images, especially in elderly patients with ocular comorbidities [10,70,96,97,98]. The presence of comorbid retinal diseases adds complexity to biomarker research in AD. Glaucoma, in particular, poses a significant challenge as it is a retinal neurodegenerative disease characterised by RNFL loss. There are hypotheses suggesting that AD may increase glaucoma risk, or that AD and glaucoma may share a common pathophysiological pathway, both contributing to retinal neurodegeneration. This overlap complicates the distinction between retinal changes associated with AD and those caused by glaucoma. Consequently, it is essential to account for glaucoma as a potential contributor to retinal thinning in AD research. To help exclude cases of glaucoma, future studies should incorporate detailed ophthalmological histories and intraocular pressure measurements. However, these methods may not identify all cases, particularly those of normal-tension glaucoma [10,53,99]. Although most studies aim to exclude patients with ARMD, the demographics of individuals with AD or preclinical AD often overlap with those at high risk for ARMD. When evaluating biomarkers that may manifest in both conditions, such as retinal amyloid deposition in drusen among ARMD patients, the ideal biomarker must effectively distinguish AD from these comorbid retinal diseases to be clinically useful [21,71,100].

Retinal biomarker profiles may be misinterpreted due to similarities between AD and other forms of cognitive impairment, such as vascular dementia and frontotemporal dementia, highlighting the necessity of being able to differentiate between these conditions [101]. Most studies using retinal imaging for AD diagnosis are cross-sectional, providing a single-time-point assessment of retinal changes. Longitudinal studies, which track retinal biomarkers over time, are essential to understanding the progression of AD and its correlation with cognitive decline. Without such data, it remains unclear whether retinal changes are early indicators of AD or secondary effects of advanced neurodegeneration. Furthermore, it is uncertain whether retinal biomarkers can reliably differentiate AD from other neurodegenerative disorders such as Parkinson’s disease or vascular dementia, underscoring the need for more extensive research.

The generalisation of current retinal imaging findings is limited by the small, homogeneous populations often studied in research. To advance the applicability of retinal biomarkers for AD detection, future research should integrate a comprehensive analysis of demographic, genetic, and social factors that may influence their expression and utility. Demographic variables such as age, sex, and ethnicity affect retinal anatomy and physiology, which may introduce variability in biomarker interpretation. Genetic predispositions, including the presence of the *Apo-E4* allele, are strongly associated with AD and may correlate with distinct retinal changes, warranting further exploration to improve diagnostic specificity. Furthermore, social determinants of health, such as socioeconomic status, access to healthcare, and education level, play a critical role in the feasibility and equity of implementing retinal biomarker-based diagnostic protocols globally [1,21,24,26]. Addressing these multifactorial influences through inclusive, longitudinal, and diverse population studies will enhance the reliability, generalizability, and accessibility of retinal biomarkers in clinical practice. Increasing participant numbers and gathering extensive data will also enable the effective application of AI in data analysis and generating new insights [1,102].

Retinal imaging faces technical and methodological issues, including a lack of standardisation across devices and protocols. Different imaging platforms can yield inconsistent results, complicating comparisons across studies. Additionally, current imaging techniques may not capture subtle changes in deeper retinal layers or detect early-stage neurodegenerative alterations, limiting the sensitivity and specificity of retinal biomarkers for AD diagnosis. Establishing a standardised framework for future studies would enhance consistency in research design and facilitate a more reliable interpretation of results and conclusions [53]. When evaluating the costs of retinal techniques for AD and the interpretation of obtained results, it is crucial to consider various factors. Although integrating AI algorithms into these techniques may increase expenses, it offers significant advantages, including improved accuracy and precision. AI can conduct more detailed analyses of retinal data, potentially leading to the detection of subtle changes associated with early AD. Consequently, the benefits of early detection, timely intervention, and enhanced treatment effectiveness would justify this investment [1]. While retinal tests show considerable promise as biomarkers for AD, they have yet to be established as standard diagnostic tools. By integrating retinal evaluations with advanced imaging techniques such as MRI, PET, and other biomarker-based approaches, we can enhance the sensitivity and specificity of AD detection. This integration could yield a comprehensive diagnostic strategy that accounts for brain and retinal pathology. Substantial evidence indicates that retinal changes can aid in diagnosing and potentially predicting AD [1]. For these methods to be fully integrated, further research and longitudinal data collection from multicentre studies are essential. Such investigations will be crucial in determining whether neuronal loss and microvascular damage in the retina of patients with MCI or the preclinical stages of AD correlate with cognitive decline and corresponding neuronal and vascular damage in the brain. By addressing these challenges and focusing on standardisation, longitudinal validation, and technological integration, retinal biomarkers can be established as essential tools for the early detection and management of AD. This approach will ultimately support the development of a comprehensive, multimodal diagnostic strategy, enhancing early detection, monitoring, and intervention strategies.

## Figures and Tables

**Table 1:** Comparison of retinal imaging techniques.

Imaging Technique	Advantages	Disadvantages
OCT	Non-invasive, detailed imaging of retinal structures. Patient-friendly. Widely accessible. Cost- and time-efficient. Tracks retinal changes and progression over time. Potential to identify at-risk individuals before the appearance of cognitive symptoms.	It does not provide comprehensive orbital images. Other retinal pathologies may influence the results.
OCTA	Non-invasive imaging of retinal vasculature. Provides detailed blood flow and perfusion information. No contrast agent is needed. High-resolution foveal imaging. Useful in diagnosing and monitoring retinal vascular changes over time.	Cannot detect all types of vascular damage. Can be affected by motion artefacts and media opacities.
Colour Fundus Photography	Non-invasive and widely accessible. Simple procedure requiring minimal training. Suitable for large-scale population screening. No need for contrast agents or invasive procedures. Automated analysis via machine learning can enhance diagnostic accuracy.	Limited specificity for AD. Less effective than OCT/OCTA in detecting early vascular alterations. Lower resolution and lack of detailed structural information. Unable to assess deeper retinal layers, missing subtle changes. Affected by factors like media opacities and poor patient fixation. Vascular changes may reflect broader conditions, not just AD-specific pathology.
Scanning Laser Ophthalmoscopy + Curcumin	Curcumin selectively binds to Aβ plaques, enabling targeted detection of AD-related retinal changes. SLO imaging is non-invasive and complements other retinal imaging techniques. Provides superior image quality for detecting subtle retinal changes. May help identify preclinical AD stages due to early Aβ aggregation.	Curcumin’s poor systemic absorption limits effectiveness, necessitating advanced formulations or delivery methods. Inconsistencies in imaging protocols and curcumin formulations hinder reproducibility and reliability. Further large-scale clinical studies are needed to confirm diagnostic accuracy and specificity for AD.

OCT: optical coherence tomography; OCTA: optical coherence tomography angiography; AD: Alzheimer’s disease; SLO: scanning laser ophthalmoscopy; Aβ: amyloid-beta.

**Table 2:** The role of OCT/OCTA in the diagnosis and monitoring of Alzheimer’s disease.

Aspect	Details
Challenge in Diagnosis	Early detection before the onset of cognitive symptoms. Family history suggests increased risk but is not sufficient for diagnosis. Need for non-invasive diagnostic tools.
OCT/OCTA as a Diagnostic Tool	RNFL thinning and GCL damage reflect neurodegenerative processes occurring in the brain. OCTA reveals alterations in retinal capillaries, offering insights into cerebrovascular health.
Retinal Biomarkers for AD	Retinal thinning, specifically RNFL reduction, is associated with amyloid plaque accumulation. Retinal biomarkers may appear before clinical symptoms. Routine imaging can monitor disease progression and evaluate therapeutic efficacy.
Vision Impairment Factors	Beta-amyloid deposits in the lens contribute to visual impairments. Retrolental amyloid deposition
Cerebrovascular Changes in the Retina in AD	OCTA shows reduced retinal blood flow and vessel density. These changes reflect cerebral hypoperfusion and white matter damage associated with AD.
Follow-Up Regime	Regular OCT/OCTA facilitates monitoring the progression of AD. Periodic scans assist in differentiating Alzheimer’s from other neurodegenerative disorders.

OCT: optical coherence tomography; OCTA: optical coherence tomography angiography; AD: Alzheimer’s disease; RNFL: retinal nerve fibre layer; GCL: ganglion cell layer.

**Table 3:** Recommendations for integrating retinal biomarkers in Alzheimer’s disease research and diagnosis.

Recommendation	Implementation Methods
Standardisation of Imaging Protocols	Establish internationally recognised standards for retinal image acquisition, processing, and analysis to enhance reproducibility and comparability across studies. Consistent protocols ensure reliability in assessing retinal biomarkers for AD.
Integration with Multimodal Biomarker Approaches	Combine retinal imaging with established diagnostic tools (CSF biomarkers, PET/CT, and MRI). Multimodal approaches provide enhanced diagnostic precision and a comprehensive understanding of AD pathology, especially in its early stages.
Longitudinal Studies with Diverse Populations	Design longitudinal studies to monitor retinal changes over time and correlate them with cognitive decline. Incorporate diverse populations to improve generalisability and reduce biases related to ethnicity, socioeconomic status, and geographic location.
Advanced Data Analysis Using AI	Implementing AI and machine learning to detect subtle retinal changes, identify complex patterns, and improve diagnostic accuracy. AI enables scalable application of retinal imaging in clinical and research settings.
Addressing Comorbid Retinal Conditions	Include detailed ophthalmological assessments for confounding conditions like glaucoma and ARMD. Develop biomarkers to distinguish AD-specific changes from other retinal pathologies.
Refining Techniques for Early Detection	Identify retinal biomarkers that manifest during the preclinical stages of AD. Early detection supports timely interventions that may alter disease progression and improve outcomes.
Economic Feasibility and Accessibility	Evaluate the cost-effectiveness of retinal imaging techniques and develop affordable, scalable technologies to ensure widespread accessibility.
Collaborative Multicentre Research	Promote collaboration among research centres to synchronise methodologies, share data, and validate findings. Multicentre studies accelerate the translation of retinal imaging research into clinical practice.
Enhanced Imaging Capabilities	Develop advanced imaging technologies to detect subtle changes in deeper retinal layers and early neurodegenerative alterations. Improved sensitivity and resolution expand the diagnostic utility of retinal biomarkers.
Educational and Training Initiatives	Establish training programmes for healthcare professionals to interpret retinal imaging results effectively and integrate these biomarkers into routine clinical practice. Education enhances the adoption and impact of these technologies on patient care.

AD: Alzheimer’s disease; CSF: cerebrospinal fluid; PET: positron emission tomography; CT: computed tomography; MRI: magnetic resonance imaging; AI: artificial intelligence; ARMD: age-related macular degeneration.

## References

[1] Yuan A., Lee C.S. (2022). Retinal Biomarkers for Alzheimer Disease: The Facts and the Future. Asia Pac. J. Ophthalmol..

[2] Kaštelan S., Bras M., Pjevac N., Bakija I., Tomic Z., PjevacKeleminic N., GverovicAntunica A. (2023). Tear Biomarkers and Alzheimer’s Disease. Int. J. Mol. Sci..

[3] Hane F.T., Robinson M., Lee B.Y., Bai O., Leonenko Z., Albert M.S. (2017). Recent Progress in Alzheimer’s Disease Research, Part 3: Diagnosis and Treatment. J. Alzheimer’s Dis..

[4] Wong W. (2020). Economic burden of Alzheimer disease and managed care considerations. Am. J. Manag. Care.

[5] Gustavsson A., Norton N., Fast T., Frölich L., Georges J., Holzapfel D., Kirabali T., Krolak-Salmon P., Rossini P.M., Ferretti M.T. (2023). Global estimates on the number of persons across the Alzheimer’s disease continuum. Alzheimer’s Dement..

[6] Kaštelan S., Pjevač N., Braš M., Đorđević V., Pjevač Keleminić N., Mezzich J.E. (2024). Person-centered care in ophthalmology: Current knowledge and perspectives. Croat. Med. J..

[7] Zhang Y., Wang Y., Shi C., Shen M., Lu F. (2021). Advances in retina imaging as potential biomarkers for early diagnosis of Alzheimer’s disease. Transl. Neurodegener..

[8] Jack C.R., Andrews J.S., Beach T.G., Buracchio T., Dunn B., Graf A., Hansson O., Ho C., Jagust W., McDade E. (2024). Revised criteria for diagnosis and staging of Alzheimer’s disease: Alzheimer’s Association Workgroup. Alzheimer’s Dement..

[9] Li C., Wang S., Xia Y., Shi F., Tang L., Yang Q., Feng J., Li C. (2024). Risk factors and predictive models in the progression from MCI to Alzheimer’s disease. Neuroscience.

[10] Aramadaka S., Mannam R., Sankara Narayanan R., Bansal A., Yanamaladoddi V.R., Sarvepalli S.S., Vemula S.L. (2023). Neuroimaging in Alzheimer’s Disease for Early Diagnosis: A Comprehensive Review. Cureus.

[11] Tanaka F., Maeda M., Kishi S., Kogue R., Umino M., Ishikawa H., Ii Y., Shindo A., Sakuma H. (2024). Updated imaging markers in cerebral amyloid angiopathy: What radiologists need to know. Jpn. J. Radiol..

[12] Patil S., Ayubcha C., Teichner E., Subtirelu R., Cho J.H., Ghonim M., Ghonim M., Werner T.J., Høilund-Carlsen P.F., Alavi A. (2025). Clinical Applications of PET Imaging in Alzheimer’s Disease. PET Clin..

[13] Quinn J.F., Gray N.E. (2024). Fluid Biomarkers in Dementia Diagnosis. Continuum.

[14] Hunter T.R., Santos L.E., Tovar-Moll F., De Felice F.G. (2025). Alzheimer’s disease biomarkers and their current use in clinical research and practice. Mol. Psychiatry.

[15] Pardo-Moreno T., Gonzalez-Acedo A., Rivas-Dominguez A., Garcia-Morales V., Garcia-Cozar F.J., Ramos-Rodriguez J.J., Melguizo-Rodriguez L. (2022). Therapeutic Approach to Alzheimer’s Disease: Current Treatments and New Perspectives. Pharmaceutics.

[16] Rampa A., Gobbi S., Belluti F., Bisi A. (2021). Tackling Alzheimer’s Disease with Existing Drugs: A Promising Strategy for Bypassing Obstacles. Curr. Med. Chem..

[17] Reiss A.B., Muhieddine D., Jacob B., Mesbah M., Pinkhasov A., Gomolin I.H., Stecker M.M., Wisniewski T., De Leon J. (2023). Alzheimer’s Disease Treatment: The Search for a Breakthrough. Medicina.

[18] Compta Y., Revesz T. (2021). Neuropathological and Biomarker Findings in Parkinson’s Disease and Alzheimer’s Disease: From Protein Aggregates to Synaptic Dysfunction. J. Park.'s Dis..

[19] Koronyo Y., Biggs D., Barron E., Boyer D.S., Pearlman J.A., Au W.J., Kile S.J., Blanco A., Fuchs D.T., Ashfaq A. (2017). Retinal amyloid pathology and proof-of-concept imaging trial in Alzheimer’s disease. JCI Insight.

[20] Chimthanawala N.M.A., Haria A., Sathaye S. (2024). Non-invasive Biomarkers for Early Detection of Alzheimer’s Disease: A New-Age Perspective. Mol. Neurobiol..

[21] Kaštelan S., Nikuseva-Martic T., Pasalic D., Antunica A.G., Zimak D.M. (2024). Genetic and Epigenetic Biomarkers Linking Alzheimer’s Disease and Age-Related Macular Degeneration. Int. J. Mol. Sci..

[22] García-Bermúdez M.Y., Vohra R., Freude K., van Wijngaarden P., Martin K., Thomsen M.S., Aldana B.I., Kolko M. (2023). Potential Retinal Biomarkers in Alzheimer’s Disease. Int. J. Mol. Sci..

[23] Liao C., Xu J., Chen Y., Ip N.Y. (2021). Retinal Dysfunction in Alzheimer’s Disease and Implications for Biomarkers. Biomolecules.

[24] Gupta V.B., Chitranshi N., den Haan J., Mirzaei M., You Y., Lim J.K., Basavarajappa D., Godinez A., Di Angelantonio S., Sachdev P. (2021). Retinal changes in Alzheimer’s disease—Integrated prospects of imaging, functional and molecular advances. Prog. Retin. Eye Res..

[25] Shi Z., Zheng H., Hu J., Jiang L., Cao X., Chen Y., Mei X., Li C., Shen Y. (2019). Retinal Nerve Fiber Layer Thinning Is Associated With Brain Atrophy: A Longitudinal Study in Nondemented Older Adults. Front. Aging Neurosci..

[26] Lin Y.F., Smith A.V., Aspelund T., Betensky R.A., Smoller J.W., Gudnason V., Launer L.J., Blacker D. (2019). Genetic overlap between vascular pathologies and Alzheimer’s dementia and potential causal mechanisms. Alzheimer’s Dement..

[27] Zheng C., Zeng R., Wu G., Hu Y., Yu H. (2024). Beyond Vision: A View from Eye to Alzheimer’s Disease and Dementia. J. Prev. Alzheimer’s Dis..

[28] Gong L., Xu R., Liu D., Lan L., Zhang B., Zhang C. (2020). The Specific Impact of Apolipoprotein E Epsilon 2 on Cognition and Brain Function in Cognitively Normal Elders and Mild Cognitive Impairment Patients. Front. Aging Neurosci..

[29] DeTure M.A., Dickson D.W. (2019). The neuropathological diagnosis of Alzheimer’s disease. Mol. Neurodegener..

[30] Soria Lopez J.A., Gonzalez H.M., Leger G.C. (2019). Alzheimer’s disease. Handbook of Clinical Neurology.

[31] Lane C.A., Hardy J., Schott J.M. (2018). Alzheimer’s disease. Eur. J. Neurol..

[32] Rostagno A.A. (2022). Pathogenesis of Alzheimer’s Disease. Int. J. Mol. Sci..

[33] Knapskog A.B., Engedal K., Selbaek G., Oksengard A.R. (2021). Alzheimer’s disease—Diagnosis and treatment. Tidsskr. Nor. Laegeforen.

[34] Ravichandran S., Snyder P.J., Alber J., Kenny M.R., Rothstein A., Brown K., Murchison C.F., Clay O.J., Roberson E.D., Arthur E. (2024). Quantifying Putative Retinal Gliosis in Preclinical Alzheimer’s Disease. Investig. Ophthalmol. Vis. Sci..

[35] London A., Benhar I., Schwartz M. (2013). The retina as a window to the brain-from eye research to CNS disorders. Nat. Rev. Neurol..

[36] Shi H., Mirzaei N., Koronyo Y., Davis M.R., Robinson E., Braun G.M., Jallow O., Rentsendorj A., Ramanujan V.K., Fert-Bober J. (2024). Identifying retinal oligomeric, citrullinated, and other tau isoforms in early and advanced AD and relations to disease status. Acta Neuropathol..

[37] Cao Q., Yang S., Wang X., Sun H., Chen W., Wang Y., Gao J., Wu Y., Yang Q., Chen X. (2024). Transport of beta-amyloid from brain to eye causes retinal degeneration in Alzheimer’s disease. J. Exp. Med..

[38] Donato L., Mordà D., Scimone C., Alibrandi S., D’Angelo R., Sidoti A. (2023). Bridging Retinal and Cerebral Neurodegeneration: A Focus on Crosslinks between Alzheimer-Perusini’s Disease and Retinal Dystrophies. Biomedicines.

[39] Ashok A., Singh N., Chaudhary S., Bellamkonda V., Kritikos A.E., Wise A.S., Rana N., McDonald D., Ayyagari R. (2020). Retinal Degeneration and Alzheimer’s Disease: An Evolving Link. Int. J. Mol. Sci..

[40] Gao R., Luo H., Yan S., Ba L., Peng S., Bu B., Sun X., Zhang M. (2024). Retina as a potential biomarker for the early stage of Alzheimer’s disease spectrum. Ann. Clin. Transl. Neurol..

[41] Shi H., Koronyo Y., Rentsendorj A., Regis G.C., Sheyn J., Fuchs D.T., Kramerov A.A., Ljubimov A.V., Dumitrascu O.M., Rodriguez A.R. (2020). Identification of early pericyte loss and vascular amyloidosis in Alzheimer’s disease retina. Acta Neuropathol..

[42] Kaštelan S., Bogadi M., Bakija I. (2022). Eyes as the Window to the Brain—A Key to the Schizophrenia Puzzle. Psychiatr. Danub..

[43] Egle M., Deal J.A., Walker K.A., Wong D.F., Sharrett A.R., Gottesman R.F. (2024). Association between retinal microvascular abnormalities and late-life brain amyloid-beta deposition: The ARIC-PET study. Alzheimer’s Res. Ther..

[44] Alber J., Bouwman F., Haan J.D., Rissman R.A., De Groef L., Koronyo-Hamaoui M., Lengyel I., Thal D.R., Alzheimer’s Association ISTAART “The Eye as a Biomarker for AD” Professional Interest Area (2024). Retina pathology as a target for biomarkers for Alzheimer’s disease: Current status, ophthalmopathological background, challenges, and future directions. Alzheimer’s Dement..

[45] Wang L., Mao X. (2021). Role of Retinal Amyloid-beta in Neurodegenerative Diseases: Overlapping Mechanisms and Emerging Clinical Applications. Int. J. Mol. Sci..

[46] Koronyo Y., Rentsendorj A., Mirzaei N., Regis G.C., Sheyn J., Shi H., Barron E., Cook-Wiens G., Rodriguez A.R., Medeiros R. (2023). Retinal pathological features and proteome signatures of Alzheimer’s disease. Acta Neuropathol..

[47] Atri A. (2019). The Alzheimer’s Disease Clinical Spectrum: Diagnosis and Management. Med. Clin. N. Am..

[48] Arvanitakis Z., Shah R.C., Bennett D.A. (2019). Diagnosis and Management of Dementia: Review. JAMA.

[49] Porsteinsson A.P., Isaacson R.S., Knox S., Sabbagh M.N., Rubino I. (2021). Diagnosis of Early Alzheimer’s Disease: Clinical Practice in 2021. J. Prev. Alzheimer’s Dis..

[50] van Oostveen W.M., de Lange E.C.M. (2021). Imaging Techniques in Alzheimer’s Disease: A Review of Applications in Early Diagnosis and Longitudinal Monitoring. Int. J. Mol. Sci..

[51] Colligris P., Perez de Lara M.J., Colligris B., Pintor J. (2018). Ocular Manifestations of Alzheimer’s and Other Neurodegenerative Diseases: The Prospect of the Eye as a Tool for the Early Diagnosis of Alzheimer’s Disease. J. Ophthalmol..

[52] van Wijngaarden P., Hadoux X., Alwan M., Keel S., Dirani M. (2017). Emerging ocular biomarkers of Alzheimer disease. Clin. Exp. Ophthalmol..

[53] Cheung C.Y., Mok V., Foster P.J., Trucco E., Chen C., Wong T.Y. (2021). Retinal imaging in Alzheimer’s disease. J. Neurol. Neurosurg. Psychiatry.

[54] Snyder P.J., Alber J., Alt C., Bain L.J., Bouma B.E., Bouwman F.H., DeBuc D.C., Campbell M.C.W., Carrillo M.C., Chew E.Y. (2021). Retinal imaging in Alzheimer’s and neurodegenerative diseases. Alzheimer’s Dement..

[55] Spaide R.F., Fujimoto J.G., Waheed N.K., Sadda S.R., Staurenghi G. (2018). Optical coherence tomography angiography. Prog. Retin. Eye Res..

[56] Attiku Y., He Y., Nittala M.G., Sadda S.R. (2021). Current status and future possibilities of retinal imaging in diabetic retinopathy care applicable to low- and medium-income countries. Indian J. Ophthalmol..

[57] Aumann S., Donner S., Fischer J., Muller F., Bille J.F. (2019). Optical Coherence Tomography (OCT): Principle and Technical Realization. High Resolution Imaging in Microscopy and Ophthalmology: New Frontiers in Biomedical Optics.

[58] Olivares Ordoñez M.A., Smith R.C., Yiu G., Liu Y.A. (2025). Retinal Microstructural and Microvascular Changes in Alzheimer Disease: A Review. Int. Ophthalmol. Clin..

[59] Doustar J., Torbati T., Black K.L., Koronyo Y., Koronyo-Hamaoui M. (2017). Optical Coherence Tomography in Alzheimer’s Disease and Other Neurodegenerative Diseases. Front. Neurol..

[60] Firmani G., Salducci M., Testa F., Covelli G.P., Sagnelli P., Lambiase A. (2024). Ocular Biomarkers in Alzheimer’s Disease: Insights into Early Detection Through Eye-Based Diagnostics—A Literature Review. Clin. Ter..

[61] Mainster M.A., Desmettre T., Querques G., Turner P.L., Ledesma-Gil G. (2022). Scanning laser ophthalmoscopy retroillumination: Applications and illusions. Int. J. Retin. Vitr..

[62] Dumitrascu O.M., Doustar J., Fuchs D.T., Koronyo Y., Sherman D.S., Miller M.S., Johnson K.O., Carare R.O., Verdooner S.R., Lyden P.D. (2024). Retinal peri-arteriolar versus peri-venular amyloidosis, hippocampal atrophy, and cognitive impairment: Exploratory trial. Acta Neuropathol. Commun..

[63] Dumitrascu O.M., Lyden P.D., Torbati T., Sheyn J., Sherzai A., Sherzai D., Sherman D.S., Rosenberry R., Cheng S., Johnson K.O. (2020). Sectoral segmentation of retinal amyloid imaging in subjects with cognitive decline. Alzheimer’s Dement..

[64] Sidiqi A., Wahl D., Lee S., Ma D., To E., Cui J., To E., Beg M.F., Sarunic M., Matsubara J.A. (2020). In vivo Retinal Fluorescence Imaging With Curcumin in an Alzheimer Mouse Model. Front. Neurosci..

[65] Khan M.A., Smith L.M. (2021). Retinal Imaging in Alzheimer’s Disease: A Review of Color Fundus Photography and Emerging Techniques. J. Alzheimer’s Dis..

[66] Mishra C., Tripathy K. (2025). Fundus Camera. StatPearls [Internet].

[67] Rim T.H., Teo A.W.J., Yang H.H.S., Cheung C.Y., Wong T.Y. (2020). Retinal Vascular Signs and Cerebrovascular Diseases. J. Neuro-Ophthalmol..

[68] Liang K., Li X., Guo Q., Ma J., Yang H., Fan Y., Yang D., Shi X., She Z., Qi X. (2024). Structural changes in the retina and serum HMGB1 levels are associated with decreased cognitive function in patients with Parkinson’s disease. Neurobiol. Dis..

[69] Tadokoro K., Yamashita T., Kimura S., Nomura E., Ohta Y., Omote Y., Takemoto M., Hishikawa N., Morihara R., Morizane Y. (2021). Retinal Amyloid Imaging for Screening Alzheimer’s Disease. J. Alzheimer’s Dis..

[70] Ashraf G., McGuinness M., Khan M.A., Obtinalla C., Hadoux X., van Wijngaarden P. (2023). Retinal imaging biomarkers of Alzheimer’s disease: A systematic review and meta-analysis of studies using brain amyloid beta status for case definition. Alzheimer’s Dement..

[71] Ge Y.J., Xu W., Ou Y.N., Qu Y., Ma Y.H., Huang Y.Y., Shen X.N., Chen S.D., Tan L., Zhao Q.H. (2021). Retinal biomarkers in Alzheimer’s disease and mild cognitive impairment: A systematic review and meta-analysis. Ageing Res. Rev..

[72] Zhang Z., Kwapong W.R., Cao L., Feng Z., Liu P., Wang R., Wu B., Zhang S. (2024). Correlation between serum biomarkers, brain volume, and retinal neuronal loss in early-onset Alzheimer’s disease. Neurol. Sci..

[73] Lian T.H., Jin Z., Qu Y.Z., Guo P., Guan H.Y., Zhang W.J., Ding D.Y., Li D.N., Li L.X., Wang X.M. (2020). The Relationship Between Retinal Nerve Fiber Layer Thickness and Clinical Symptoms of Alzheimer’s Disease. Front. Aging Neurosci..

[74] Zhao B., Yan Y., Wu X., Geng Z., Wu Y., Xiao G., Wang L., Zhou S., Wei L., Wang K. (2023). The correlation of retinal neurodegeneration and brain degeneration in patients with Alzheimer’s disease using optical coherence tomography angiography and MRI. Front. Aging Neurosci..

[75] Wang R., Wu X., Zhang Z., Cao L., Kwapong W.R., Wang H., Tao W., Ye C., Liu J., Wu B. (2023). Retinal ganglion cell-inner plexiform layer, white matter hyperintensities, and their interaction with cognition in older adults. Front. Aging Neurosci..

[76] Moussa M., Falfoul Y., Nasri A., El Matri K., Kacem I., Mrabet S., Chebil A., Gharbi A., Gouider R., El Matri L. (2023). Optical coherence tomography and angiography in Alzheimer’s disease and other cognitive disorders. Eur. J. Ophthalmol..

[77] Dehghani C., Frost S., Jayasena R., Masters C.L., Kanagasingam Y. (2018). Ocular Biomarkers of Alzheimer’s Disease: The Role of Anterior Eye and Potential Future Directions. Investig. Ophthalmol. Vis. Sci..

[78] Moncaster J.A., Moir R.D., Burton M.A., Chadwick O., Minaeva O., Alvarez V.E., Ericsson M., Clark J.I., McKee A.C., Tanzi R.E. (2022). Alzheimer’s disease amyloid-beta pathology in the lens of the eye. Exp. Eye Res..

[79] Frost S., Kanagasingam Y., Sohrabi H., Vignarajan J., Bourgeat P., Salvado O., Villemagne V., Rowe C.C., Macaulay S.L., Szoeke C. (2013). Retinal vascular biomarkers for early detection and monitoring of Alzheimer’s disease. Transl. Psychiatry.

[80] Sánchez D., Castilla-Marti M., Rodríguez-Gómez O., Valero S., Piferrer A., Martínez G., Martínez J., Serra J., Moreno-Grau S., Hernández-Olasagarre B. (2018). Usefulness of peripapillary nerve fiber layer thickness assessed by optical coherence tomography as a biomarker for Alzheimer’s disease. Sci. Rep..

[81] Carazo-Barrios L., Archidona-Arranz A., Claros-Ruiz A., Garcia-Basterra I., Garzon-Maldonado F.J., Serrano-Castro V., Gutierrez-Bedmar M., Barbancho M.A., De la Cruz Cosme C., Garcia-Campos J.M. (2021). Correlation between retinal nerve fibre layer thickness and white matter lesions in Alzheimer’s disease. Int. J. Geriatr. Psychiatry.

[82] Salvadó G., Horie K., Barthélemy N.R., Vogel J.W., PichetBinette A., Chen C.D., Aschenbrenner A.J., Gordon B.A., Benzinger T.L.S., Holtzman D.M. (2024). Disease staging of Alzheimer’s disease using a CSF-based biomarker model. Nat. Aging.

[83] Bulut M., Kurtulus F., Gozkaya O., Erol M.K., Cengiz A., Akidan M., Yaman A. (2018). Evaluation of optical coherence tomography angiographic findings in Alzheimer’s type dementia. Br. J. Ophthalmol..

[84] Eraslan Boz H., Koçoğlu K., Akkoyun M., Tüfekci I.Y., Ekin M., Akdal G. (2024). Visual search in Alzheimer’s disease and amnestic mild cognitive impairment: An eye-tracking study. Alzheimer’s Dement..

[85] Hussain A., Sheikh Z., Subramanian M. (2023). The Eye as a Diagnostic Tool for Alzheimer’s Disease. Life.

[86] Li Y., Liu X., Liu Q., Wang Y., Liu C., Chen F. (2025). Green Synthesis of Alzheimer’s Disease Probes Aftobetin and Analogues Enabled by Flow Technology and Heterogeneous Photocatalysis. ChemSusChem.

[87] Sharma M., Pal P., Gupta S.K. (2024). Deciphering the role of miRNAs in Alzheimer’s disease: Predictive targeting and pathway modulation—A systematic review. Ageing Res. Rev..

[88] Lin P., Xu J., Yang F., Li D., Zhang R., Jiang Y., Zheng T. (2024). Elevated concentrations of amyloid-β oligomers and their proapoptotic effects on age-related cataract. FASEB J..

[89] Romaus-Sanjurjo D., Regueiro U., López-López M., Vázquez-Vázquez L., Ouro A., Lema I., Sobrino T. (2022). Alzheimer’s Disease Seen through the Eye: Ocular Alterations and Neurodegeneration. Int. J. Mol. Sci..

[90] Majeed A., Marwick B., Yu H., Fadavi H., Tavakoli M. (2021). Ophthalmic Biomarkers for Alzheimer’s Disease: A Review. Front. Aging Neurosci..

[91] Rahman M.M., Mim S.A., Islam M.R., Parvez A., Islam F., Uddin M.B., Rahaman M.S., Shuvo P.A., Ahmed M., Greig N.H. (2022). Exploring the Recent Trends in Management of Dementia and Frailty: Focus on Diagnosis and Treatment. Curr. Med. Chem..

[92] Gao Y., Wang R., Mou K., Zhang Y., Xu H., Liu Y., Yang F., Gao Y., Wang X., Bao L. (2024). Association of outer retinal and choroidal alterations with neuroimaging and clinical features in posterior cortical atrophy. Alzheimer’s Res. Ther..

[93] Ibrahim Y., Xie J., Macerollo A., Sardone R., Shen Y., Romano V., Zheng Y. (2023). A Systematic Review on Retinal Biomarkers to Diagnose Dementia from OCT/OCTA Images. J. Alzheimer’s Dis. Rep..

[94] Xu Y., Aung H.L., Hesam-Shariati N., Keay L., Sun X., Phu J., Honson V., Tully P.J., Booth A., Lewis E. (2024). Contrast Sensitivity, Visual Field, Color Vision, Motion Perception, and Cognitive Impairment: A Systematic Review. J. Am. Med. Dir. Assoc..

[95] Aiello Bowles E.J., Crane P.K., Walker R.L., Chubak J., LaCroix A.Z., Anderson M.L., Rosenberg D., Keene C.D., Larson E.B. (2019). Cognitive Resilience to Alzheimer’s Disease Pathology in the Human Brain. J. Alzheimer’s Dis..

[96] Carazo-Barrios L., Cabrera-Maestre A., Alba-Linero C., Gutierrez-Bedmar M., Garzon-Maldonado F.J., Serrano V., de la Cruz-Cosme C., Garcia-Casares N. (2023). Retinal Neurodegeneration Measured With Optical Coherence Tomography and Neuroimaging in Alzheimer Disease: A Systematic Review. J. Neuro-Ophthalmol..

[97] Chan V.T.T., Sun Z., Tang S., Chen L.J., Wong A., Tham C.C., Wong T.Y., Chen C., Ikram M.K., Whitson H.E. (2019). Spectral-Domain OCT Measurements in Alzheimer’s Disease: A Systematic Review and Meta-analysis. Ophthalmology.

[98] Gaire B.P., Koronyo Y., Fuchs D.T., Shi H., Rentsendorj A., Danziger R., Vit J.P., Mirzaei N., Doustar J., Sheyn J. (2024). Alzheimer’s disease pathophysiology in the Retina. Prog. Retin. Eye Res..

[99] den Haan J., Verbraak F.D., Visser P.J., Bouwman F.H. (2017). Retinal thickness in Alzheimer’s disease: A systematic review and meta-analysis. Alzheimer’s Dement..

[100] Jabbehdari S., Oganov A.C., Rezagholi F., Mohammadi S., Harandi H., Yazdanpanah G., Arevalo J.F. (2024). Age-related macular degeneration and neurodegenerative disorders: Shared pathways in complex interactions. Surv. Ophthalmol..

[101] Chalkias E., Topouzis F., Tegos T., Tsolaki M. (2021). The Contribution of Ocular Biomarkers in the Differential Diagnosis of Alzheimer’s Disease versus Other Dementia and Future Prospects. J. Alzheimer’s Dis..

[102] Abraham A.G., Guo X., Arsiwala L.T., Dong Y., Sharrett A.R., Huang D., You Q., Liu L., Lujan B.J., Tomlinson A. (2021). Cognitive decline in older adults: What can we learn from optical coherence tomography (OCT)-based retinal vascular imaging?. J. Am. Geriatr. Soc..

